# Probing of Exosites Leads to Novel Inhibitor Scaffolds of HCV NS3/4A Proteinase

**DOI:** 10.1371/journal.pone.0040029

**Published:** 2012-07-02

**Authors:** Sergey A. Shiryaev, Anton V. Cheltsov, Alex Y. Strongin

**Affiliations:** 1 Inflammatory and Infectious Disease Center, Sanford-Burnham Medical Research Institute, La Jolla, California, United States of America; 2 R&D Department, Q-MOL L.L.C., San Diego, California, United States of America; University of Chicago, United States of America

## Abstract

**Background:**

Hepatitis C is a treatment-resistant disease affecting millions of people worldwide. The hepatitis C virus (HCV) genome is a single-stranded RNA molecule. After infection of the host cell, viral RNA is translated into a polyprotein that is cleaved by host and viral proteinases into functional, structural and non-structural, viral proteins. Cleavage of the polyprotein involves the viral NS3/4A proteinase, a proven drug target. HCV mutates as it replicates and, as a result, multiple emerging quasispecies become rapidly resistant to anti-virals, including NS3/4A inhibitors.

**Methodology/Principal Findings:**

To circumvent drug resistance and complement the existing anti-virals, NS3/4A inhibitors, which are additional and distinct from the FDA-approved telaprevir and boceprevir α-ketoamide inhibitors, are required. To test potential new avenues for inhibitor development, we have probed several distinct exosites of NS3/4A which are either outside of or partially overlapping with the active site groove of the proteinase. For this purpose, we employed virtual ligand screening using the 275,000 compound library of the Developmental Therapeutics Program (NCI/NIH) and the X-ray crystal structure of NS3/4A as a ligand source and a target, respectively. As a result, we identified several novel, previously uncharacterized, nanomolar range inhibitory scaffolds, which suppressed of the NS3/4A activity *in vitro* and replication of a sub-genomic HCV RNA replicon with a luciferase reporter in human hepatocarcinoma cells. The binding sites of these novel inhibitors do not significantly overlap with those of α-ketoamides. As a result, the most common resistant mutations, including V36M, R155K, A156T, D168A and V170A, did not considerably diminish the inhibitory potency of certain novel inhibitor scaffolds we identified.

**Conclusions/Significance:**

Overall, the further optimization of both the *in silico* strategy and software platform we developed and lead compounds we identified may lead to advances in novel anti-virals.

## Introduction

Hepatitis C is a treatment-resistant disease with over 200 million people infected worldwide. Over 80% of infected patients develop chronic hepatitis. The HCV genome is a single-stranded RNA molecule with positive polarity that is ∼9,600 nucleotides in length. After infection of the host cell and liberation of the RNA genome from the protecting virus particle, the viral RNA is translated into a multi-domain polyprotein that is proteolytically cleaved into ten products [1]. The structural proteins are then used to assemble new virus particles, while the non-structural (NS) proteins participate in the replication of the viral genome. In the course of RNA replication, the viral genome is used as a template for the synthesis of negative-strand RNA, which next acts as a template for the production of positive-strand RNA. Replication is catalyzed by the NS3 helicase and the NS5B RNA-dependent RNA polymerase. The helicase represents the C-terminal portion of the NS3 protein. The NS3 helicase unwinds in an ATP-dependent manner double-stranded RNA into single strands (reviewed by Penin et al [2]).

The chymotrypsin-like NS3 serine proteinase (NS3/4A) represents the N-terminal portion of the NS3 protein. NS3/4A cleaves the viral polyprotein precursor at the NS3/NS4A, NS4A/NS4B, NS4B/NS5A and NS5A/NS5B junction regions. The individual NS3 proteinase domain, however, is inactive. For cleavage activity *in vitro* and *in vivo*, the NS3 domain requires the NS4A co-factor [3]. NS4A is a 54 residue amphipathic protein, with a hydrophobic N-terminus and a hydrophilic C-terminus. When complexed with NS4A, the NS3/4A domain is rearranged leading to the proper alignment of His-57, Asp-81, and Ser-139 of the catalytic triad. NS3/4A exhibits a Zn-binding site that serves a structural role and that is coded by the three Cys residues (Cys-97, -99 and -145) and His-149 [4]. The NS3/4A active site is positioned between two β-barrel domains and in a shallow groove that contacts long peptide substrates by multiple weak interactions [3], [5]. The shallow active site groove allows minor structural modifications to interfere with substrate binding, promoting resistance.

Because NS5B, the RNA-dependent RNA polymerase, misincorporates bases at a high rate, HCV constantly mutates as it replicates. The process of constant mutation leads to heterogeneous viral populations and multiple quasispecies of HCV in infected patients [6], [7]. Mutations in the viral genome cause a rapid emergence of HCV genotypes which resist therapeutic intervention and help the virus to evade both the host's immune response and anti-virals [8]. As patients begin treatment, the selective pressures of anti-virals will favor drug resistant quasispecies. Mutations that confer the most severe resistance in the clinic occur where inhibitors protrude from the consensus volume defining the substrate envelope, as these changes selectively weaken inhibitor binding without compromising the substrate binding [9]. Both FDA-approved boceprevir (SCH503034) and telaprevir (VX-950) exhibit a ketoamide moiety with the catalytic serine nucleophile and these inhibitors generate a covalent, albeit reversible, enzyme-inhibitor complex [10], [11], [12]. Additional NS3/4A-targeting compounds, non-covalent reversible peptidomimetic macrocycle inhibitors such as TMC435350, MK-7009, ITMN-191, BILN-2061, BMS-791325, GS-9256 and ABT-450, have also been a subject of extensive evaluation and clinical testing in the recent years (reviewed in Halfon and Locarnini [13]). These macrocyclic inhibitors exhibit an overlapping, albeit distinct, resistance profile compared with FDA-approved boceprevir and telaprevir ketoamides [9], [11], [14], [15].

Because of its functional importance in the HCV life cycle, NS3/4A is an attractive anti-viral drug target. The current inhibitors can be roughly divided into two classes, macrocyclic and linear, peptidomimetic α-ketoamide derivatives. Peptidomimetic macrocyclic ciluprevir that non-covalently binds the NS3/4A active site failed clinical trials because of its cardiotoxicity [16], [17]. In turn, the linear peptidomimetic α-ketoamides, telaprevir and boceprevir, that bind covalently, albeit reversibly, to the active site Ser-139, have recently been approved by the FDA for clinical use. To compensate for the shallow active site groove architecture both α-ketoamides exploit interactions with catalytically non-essential amino acid residues. This results in exhibiting of a low genetic barrier for viral resistance. The association of telaprevir with NS3/4A involves a non-covalent binding step (*K_I_*≈6 µM). This step is followed by a fast covalent bond formation resulting to the improved *K_I_* value of ≈40 nM [18]. Multiple non-essential residue mutations, including, but not limited to A156F/T/V, R155K/T/Q and V36A, may rapidly lead to the telaprevir-resistant HCV, a phenomenon that has already been reported using replicon studies and murine models [14], [19] and, most importantly, has already been observed clinically at frequencies of 5 to 20% of the total virus population and as early as the second day after treatment initiation ([20], [21], [22], [23] and comprehensively reviewed in [13], [24], [25], [26], [27], [28], [29]).

To this end, we have previously demonstrated that the functional activity of the structurally similar NS2B-NS3 two-component proteinase of West Nile virus (WNV) is efficiently repressed by small molecule allosteric inhibitors [30]. Here, we employ a similar strategy to design and then test the inhibitory potency of the inhibitors that target three distinct exosites in the NS3/4A molecule. As a result, we identified novel, previously uncharacterized inhibitory scaffolds that specifically target HCV NS3/4A and the efficacy of which is not significantly affected by several common resistance mutations.

## Results

### Docking sites in NS3/4A

**Figure 1:**
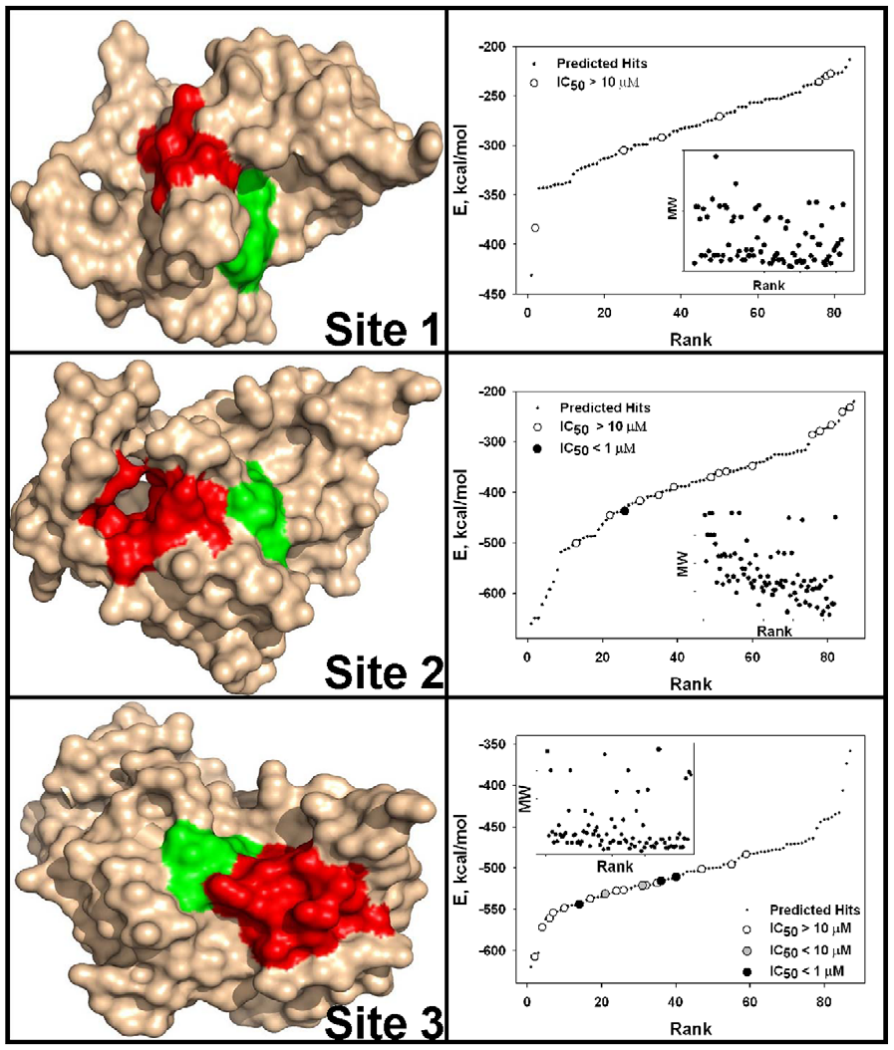
Three docking sites in NS3/4A and VLS of the NCI/DTP compound library. Left panels, positions of docking sites 1, 2 and 3 in the PDB 3EYD X-ray structure of NS3/4A, surface model. The catalytic triad (His-57, Asp-81, and Ser-139) is green. Docking sites 1, 2 and 3 are red. Right panels, VLS of the 275,000-compound NCI library against docking sites 1, 2 and 3. VLS led to identification of the top 84, 87 and 88 hits, from which 7, 15 and 18 available compounds for sites 1, 2 and 3, respectively, were tested in the NS3/4A inhibitory assays. Compounds were ranked according to their relative binding energy. Black, grey and open circles correspond to the tested compounds with the IC_50_ values below 1 µM, below 10 µM and above 10 µM, respectively. Predicted (but untested) hits are shown as small back dots. E, relative binding energy. Inset, relations between the molecular weight (MW) and ranking of the ligands.

Three sites in the NS3 proteinase domain, which are distinct from the active site groove, were specifically selected for protein-ligand docking. Selection of docking site 1 was based on the PDB 3EYD structure [3]. This site was defined as a 10 Å sphere centered at Val-26 of chain D (Fig. 1). In the PDB 3EYD structure, docking site 1 represents the surface area of the NS3 proteinase domain that is in contact with NS4A. The NS4A Val-26 residue that we used as a geometric center for the docking site is adjacent to the highly conserved Ile-25. Ile-25 directly interacts with the NS3 protease domain. Based on these structural and functional parameters of NS3/4A, we hypothesized that small molecules capable of interacting with docking site 1 may compete with the NS4A co-factor binding with the NS3 proteinase domain and, consequently, with the formation of the catalytically potent NS3/4A. Chains B, C and D were then deleted from the structure and chain A alone was used in virtual ligand screening (VLS).

**Figure 2:**
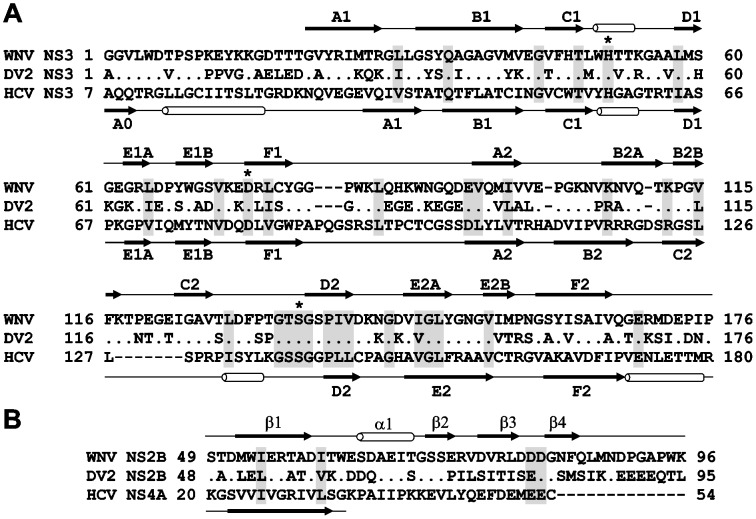
Structural similarity of the flaviviral NS3 proteinases. (A) Sequence alignment of NS3 proteinases. Asterisks mark the catalytic triad. Identical and homologous residue positions are shaded gray. (B) Sequence alignment of the flaviviral WNV and DV2 NS2B and HCV NS4A (PDB 3EYD) co-factors. Dots indicate identical residues. Secondary structure elements above and below the sequences are for WNV NS2B-NS3 proteinase (PDB 2IJO) and HCV NS3/4A (PDB 3EYD), respectively. The secondary structure of the minimal, 14-residue, NS4A co-factor required for activation of the NS3 proteinase *in vitro* is shown. WNV, West Nile virus. DV2, Dengue virus type 2.

Docking site 2 was selected because of its pocket-like shape. This pocket becomes identifiable in chain C when the NS4A co-factor is removed from the PDB 3EYD NS3/4A structure. Site 2 was defined as a 10 Å sphere centered at Val-23 of chain B. Chains A, B and D were then deleted while chain C alone was used for VLS (Fig. 1). Docking site 3 was specifically selected for targeting because its location in the NS3/4A structure is similar to that in the WNV NS2B-NS3 proteinase structure and because targeting of this site has led us to the discovery of efficient allosteric inhibitors of the WNV proteinase [30] (Fig. 2). Docking site 3 was defined as a 10 Å sphere centered at Val-167 of chain C. Chain C alone was used in VLS while chains A, B and D were deleted (Fig. 1).

### VLS and *in vitro* validation of the hits

The diverse 275,000 compound NCI database was screened using the Q-MOL software against docking sites 1, 2 and 3 of the atomic resolution structure of NS3/4A (PDB 3EYD). As a result, 84, 87 and 88 hits were identified from sites 1, 2 and 3, respectively. A cumulative Gaussian distribution of ligand rankings suggested that VLS performed most efficiently for docking site 3 compared to sites 1 and 2 (Fig. 1). The available 7, 15 and 18 ligands for sites 1, 2 and 3, respectively, were ordered from the NCI/DTP and analyzed further.

The inhibitory potency of the available compounds was then directly tested in the cleavage reactions *in vitro* using the recombinant NS3/4A and acetyl-Asp-Glu-Asp(EDANS)-Glu-Glu-Abu-ψ-[COO]-Ala-Ser-Lys(DABSYL)-NH_2_ (Ac-DE-D(Edans)-EE-Abu-ψ-[COO]-AS-K(Dabsyl)-NH_2_) as a substrate. These tests demonstrated that the site 1 ligands did not perform as potent inhibitors of the catalytic activity of NS3/4A. Only a few of these compounds exhibited the IC_50_ value in a 10–100 µM range. In contrast, compound **2** against docking site 2 performed as a 300 nM inhibitor of NS3/4A while the IC_50_ value of the multiple additional compounds were in a 10–100 µM range. From the 18 compounds for docking site 3, three compounds (**1**, **3** and **5**) displayed IC_50_ values in the 180–380 nM range. Two additional compounds to site 3 exhibited the IC_50_ in a 4.5–6.5 µM range (Tables 1 and S1).

**Table 1:** Inhibitors of HCV NS3/4A.

Compound	NCI identifier	NS3/4A docking site	NS3/4A NS2B-NS3 Furin				HCV replicon			
				WNV	DV2					
			IC_50_, µM				IC_50_, µM	IC_90_, µM	CC_50_, µM	CC_90_, µM
**1**	NSC704342	3	0.183	21.87	19.70	>100	>100	>100	>100	>100
**2**	NSC713288	2	0.3	>100	>100	>100	10.02	25.19	9.99	22.06
**3**	NSC724526	3	0.36	18.25	34.48	>100	>100	>100	>100	>100
**4**	NSC320254	3	0.36	3.7	14.33	12.76	27.99	99.4	>100	>100
**5**	NSC724527	3	0.38	23.74	49.43	>100	>100	>100	>100	>100
**6**	NSC724525	3	0.95	12.74	20.59	66.79	>100	>100	>100	>100
**7**	NSC716899	3	1.25	39.09	>100	>100	9.95	54.4	86.78	96.33
**8**	NSC637712	3	1.92	21.77	38.80	26.97	13.6	19.2	14.23	26.61

NS3/4A activity was measured using Ac-DE-D(Edans)-EE-Abu-ψ-[COO]-AS-K(Dabsyl)-NH_2_ as a peptide substrate. The activity of furin and both WNV and DV2 NS2B-NS3 proteinases was measured using pyroglutamic acid-Arg-Thr-Lys-Arg-7-amino-4-methylcoumarin as a substrate. IC_50_ and IC_90_, compound concentrations, which reduced viral replication by 50% and 90%, respectively. CC_50_ and CC_90_, compound concentrations, which reduced cell viability by 50% and 90%, respectively. >100, determined values exceeded 100 µM. Refer to Table S1 for the compound structures.

### SAR optimization

**Figure 3:**
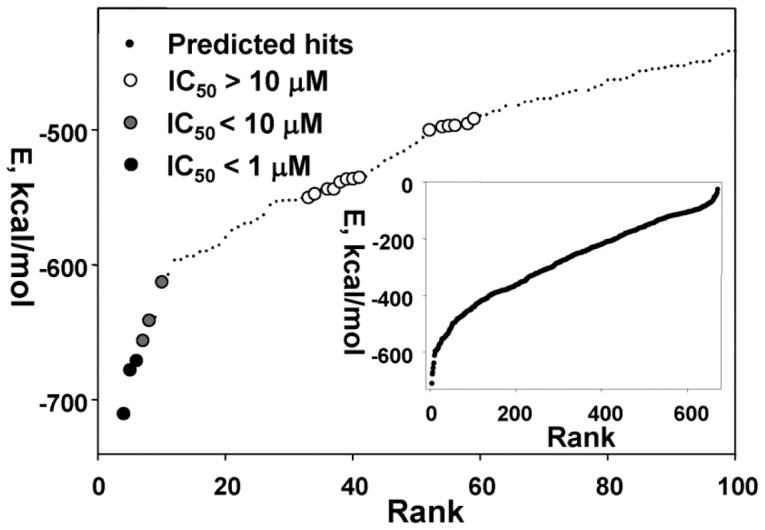
VLS and SAR optimization of the focused compound sub-library. The 665-compound sub-library was docked into docking site 3 of NS3/4A. Compounds were ranked according to their relative binding energy (inset, a complete ranking curve, each dot represents a single compound). The screening led to the identification of the top 100 compounds with the lowest binding energy. Compounds were visually inspected and the 20 available compounds were ordered from the NCI/DTP for the follow-up *in vitro* activity tests. Black, grey and open circles correspond to the tested compounds with the IC_50_ values below 1 µM, below 10 µM and above 10 µM, respectively. Predicted (but untested) hits are shown as small back dots. E, relative binding energy.

**Figure 4:**
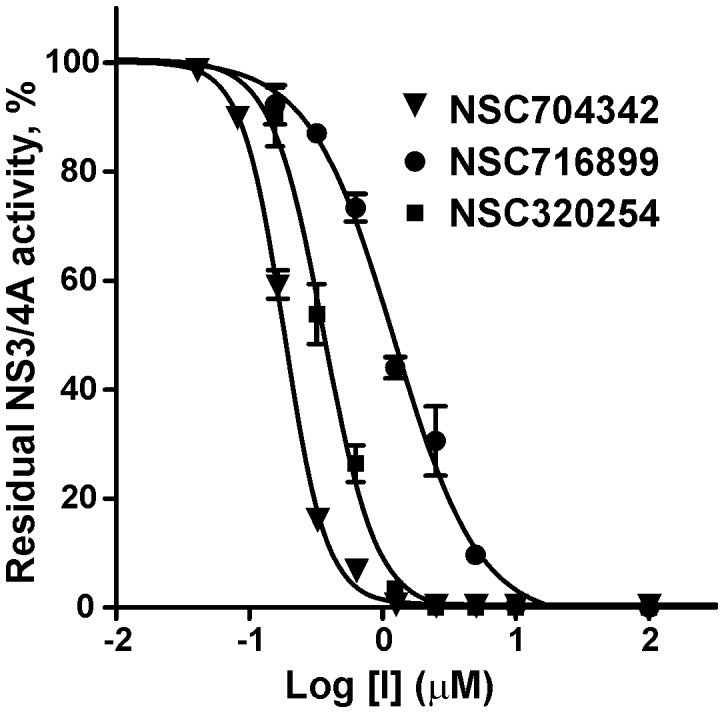
Selected compounds efficiently inhibit the catalytic activity of NS3/4A: representative dose-response curves. Before the addition of the Ac-DE-D(Edans)-EE-Abu-ψ-[COO]-AS-K(Dabsyl)-NH_2_ substrate (10 µM), NS3/4A (10 nM) was co-incubated for 30 min at ambient temperature with increasing concentrations of compounds **1, 4** and **7**. The residual activity was then monitored continuously at λ_ex_ = 355 nm and λ_em_ = 500 nm to determine the initial velocity of the reactions. The initial velocity was calculated as a percentage of residual activity versus the untreated proteinase (control). Refer to Table S1 for the compound structures.

To identify additional, structurally similar scaffolds in the NCI/DTP database and to perform scaffold hopping, we employed *in silico* SAR optimization using compounds **1**, **3** and **5** as seeds. The Tanimoto distance was as used as a chemical similarity measure of the novel compounds relative to the seeds [31], [32], [33]. For each seed structure, 250 close derivatives were selected from the NCI/DTP database. The full-atom ligand structures of the resulting 750-compound focused sub-library were then minimized using the Q-MOL minimization protocol. The structures of 665 compounds were successfully minimized and next re-docked into site 3 (Fig. 3). The 100 top compounds with the lowest binding energy were visually inspected and the 20 available compounds were ordered from the NCI/DTP for follow-up *in vitro* activity tests. From these 20 novel compounds, two ligands (compounds **4** and **6**) exhibited the IC_50_ values below 1 µM while the IC_50_ values of compounds **7, 8** and **10** were below 10 µM (Tables 1 and S1). All of the selected compounds inhibit assay 100% at the highest concentrations tested. The representative dose-response curves, which are used to calculate the compound inhibitory parameters, including the IC_50_ values, are shown in Fig. 4.

### Cross-reactivity studies

To determine their off-target activity, compounds **1–8** were tested against homologous WNV and DV NS2B-NS3 serine proteases and furin (Table 1). Compound **2** against docking site 2 did not demonstrate any noticeable off-target activity. Compounds **1**, **3** and **4** against site 3 were also significantly more selective in inhibiting HCV NS3/4A as compared to both flaviviral proteinases and furin. These data provide evidence for selectivity of the ligands and also demonstrate that the efficient inhibition of NS3/4A is not a result of protein aggregation by the compounds.

### Inhibition of HCV sub-genomic replicon

To support our *in vitro* data, the inhibition of HCV RNA replication was then measured using human hepatocarcinoma Huh7 ET cells. These cells express a sub-genomic HCV RNA replicon with a stable luciferase reporter. The replicon contains the 5′-end Internal Ribosome Entry Site (IRES) of HCV which drives the production of a firefly luciferase, ubiquitin, and neomycin phosphotransferase (Neo) fusion protein. Ubiquitin cleavage releases the luciferase and Neo proteins. The IRES element encephalomyocarditis virus controls the translation of the HCV structural proteins NS3-NS5 in the replicon. The NS3 protein cleaves the synthesized HCV polyprotein to release the mature NS3, NS4A, NS4B, NS5A and NS5B proteins that are required for HCV replication. The authentic 3′-end non-translating region of HCV is localized at the 3′-end of the replicon [34]. As a result, the activity of the luciferase reporter is directly proportional to HCV RNA levels and positive control antiviral compounds behave comparably using either luciferase or RNA endpoints.

The compounds we tested did not inhibit enzyme activity of luciferase (data not shown). Our cell-based tests, however, demonstrated that the low µM concentrations of compounds **4** and **7** were capable of repressing the intracellular luciferase reporter activity (Table 1). Thus, it is highly likely that the inhibitory hits, including compounds **4** and **7**, directly affected the replicon-encoded intracellular NS3/4A activity that is essential for processing the HCV polyprotein.

### Protein-ligand modeling

**Figure 5:**
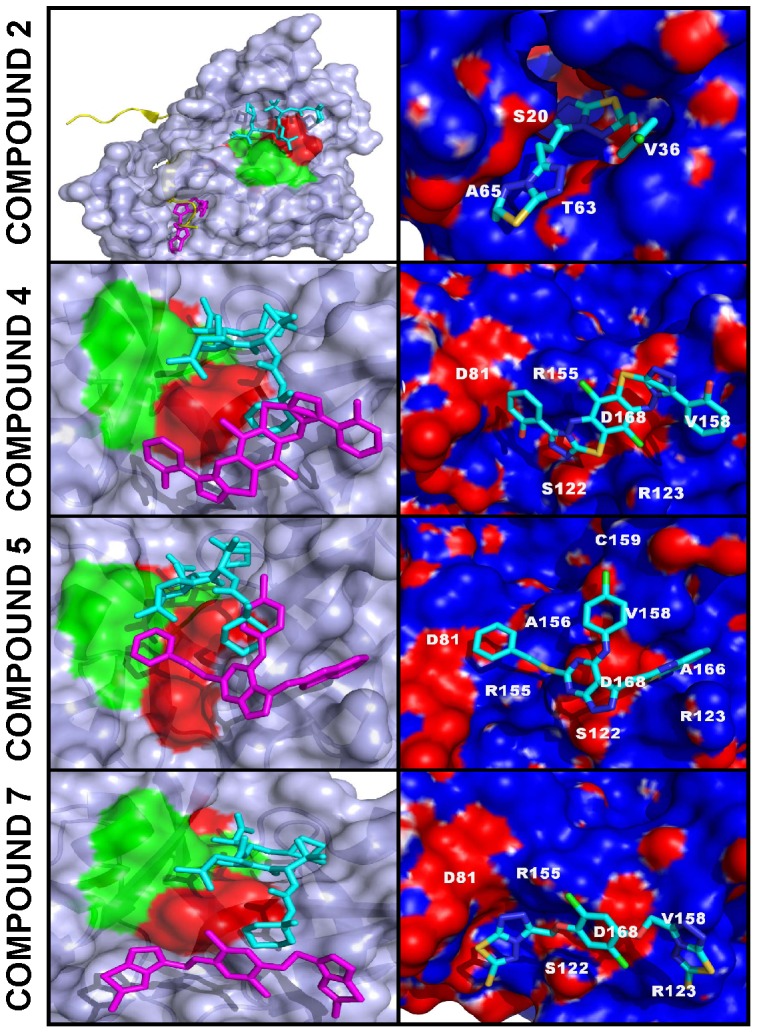
Models of NS3/4A-inhibitor complexes. Left panels, NS3/4A is shown as a surface model. Docked compounds **2, 4, 5** and **7** are shown as stick models (magenta). Mutant residue positions, which confer resistance to telaprevir and boceprevir [37], [38], [39], [40], [41], [42], are red. Catalytic triad residues (His-57, Asp-81, and Ser-139) are green. The superimposed co-crystallized boceprevir derivative (1R,2S,5S)-N-[(1S)-3-amino-1-(cyclobutylmethyl)-2,3-dioxopropyl]-6,6-dimethyl-3-{3-methyl-N-[(1-methylcyclohexyl)carbamoyl]-L-valyl}-3-azabicyclo[3.1.0]hexane-2-carboxamide (PDB 3LOX) is shown as a stick model (cyan). In the compound 2 panel the NS2B cofactor is yellow. Right panels. Close-up of the binding modes of compounds **2,** **4, 5** and **7**. NS3/4A is shown as a molecular surface model colored by electrostatic potential. The latter was corrected for solvation using the Poisson-Boltzmann equation [73], [74]. Docked compounds **2, 4, 5** and **7** are shown as stick models colored by the chemical element type. Amino acid numbering corresponds to that of PDB 3EYD. The figures were created using Pymol.

To visualize the interactions between the compounds **2, 4, 5** and **7** with docking sites 2 and 3 of NS3/4A, we modeled the inhibitor-proteinase complexes using the PDB 3EYD chain C as a template [35] (Fig. 5). PDB 3LOX [36] was superimposed with our models to demonstrate the binding mode of the boceprevir derivative relative to our novel inhibitors. Our results suggest that in contrast with boceprevir our inhibitors do not directly interact with the catalytic site of NS3/4A. According to our modeling, compound **2** is predicted to fit well into the pocket-like site 2 and to be proximal to Ser-20, Val-36, Thr-63 and Ala-65 of the NS3 domain. The binding site of compound **2** is distant from the catalytic triad and also from the residues which confer resistance to telaprevir and boceprevir.

Compounds **4** and **7** are likely to localize in a vicinity of Ser-122, Arg-123, Val-158 and Asp-168 from docking site 3, but not with Arg-155, an essential residue for binding both telaprevir and boceprevir (Fig. 5) (reviewed in [37]). The structurally distinct compound **5** is likely to localize near Arg-123, Val-158, Ala-166, Asp-168 and active site Asp-81. We cannot, however, exclude that Arg-155 and Ala-156, both of which are important for the efficient inhibition of NS3/4A by telaprevir and boceprevir [38], [39], [40], [41], [42], are also engaged in the interactions with compound **5.** The chlorinated benzene ring of compound **5** is likely to be at a short, ∼3 Å, distance from Cys-159. Because a chlorine atom directly attached to the benzene ring is normally chemically inert, we do not expect, however, that under physiological conditions compound **5** in its current form is capable of forming a covalent bond with the Cys-159 sulfo group.

### Selected compounds inhibit drug-resistant NS3/4A mutants

To determine if the compounds are active against the most common NS3/4A resistance mutations, we compared the inhibitory potency of compounds **1–8** against the NS3/4A mutants (V36M, R155K, A156T, D168A and V170A) and the wild-type NS3/4A using NS3/4A activity assay. For this purpose, the purified V36M, R155K, A156T, D168A and V170A mutant constructs were co-incubated with compounds **1–8** and the residual catalytic activity was then measured using Ac-DE-D(Edans)-EE-Abu-ψ-[COO]-AS-K(Dabsyl)-NH_2_) as a substrate.

The resistant mutations V36M, R155K, A156T and V170A are known to confer reduced sensitivity to boceprevir and telaprevir [9], [10], [11], [14], [19], [43]. Some mutations also affect, albeit differentially, the efficacy of macrocyclic inhibitors of the NS3/4A catalytic activity, such as BILN-2061, TMC435350, MK-7009, and ITMN-191 [14], [15], [16], [44], [45], [46], [47], [48]. The IC_50_ values of compounds **1–8** we recorded suggested that the resistance mutations significantly affected certain compounds while a few compounds including **6** and **7** largely retained their inhibitory potency in a way that is more favorable compared with that of boceprevir and telaprevir (Table 2). In general, our experimental results agree well with our docking and modeling studies. For instance, the potency of compound **5** was most significantly affected the D168A mutation. This effect is consistent with the predicted binding mode of compound **5** (Fig. 5). Intriguingly, the presence of a thiazole-thione group (compound **6**) relative to a triazole-thione group (compound **3**) appeared to contribute favorably to the inhibitor's ability to overcome the resistance mutations. In contrast with boceprevir and telaprevir, the efficacy of compound **6** and, especially compound **7**, was not affected by the A156T resistance mutation. We conclude that our proof-of-principle data imply that the fine-tuned and optimized derivatives of these novel inhibitory scaffolds could be developed as supplements to the existing anti-viral therapy in HCV patients with certain drug-resistant HCV quasispecies.

**Table 2:** Effects of resistance mutations on the inhibitory activity of compounds 1–8.

Compound NCI identifier	NS3/4A											
	Docking site	WT	V36M		R155K		A156T		D168A		V170A	
		IC_50_, µM	IC_50_, µM	FC	IC_50_, µM	FC	IC_50_, µM	FC	IC_50_, µM	FC	IC_50_, µM	FC
**1** NSC704342	3	0.183	0.26	1.4	1.75	9.6	0.72	3.93	0.25	1.4	0.40	2.2
**2** NSC713288	2	0.30	1.53	5.1	5.84	19.5	17.27	57.57	4.45	14.8	1.80	6
**3** NSC724526	3	0.36	1.67	4.6	5.53	15.4	16.25	45.14	8.40	23.3	1.97	5.5
**4** NSC320254	3	0.36	0.34	0.9	3.73	10.4	6.54	18.17	3.35	9.3	0.80	2.2
**5** NSC724527	3	0.38	1.07	2.8	9.64	25.4	18.60	48.95	12.69	33.4	6.29	16.6
**6** NSC724525	3	0.95	0.99	1.04	2.29	2.4	3.08	3.24	1.21	1.3	0.93	0.98
**7** NSC716899	3	1.25	1.40	1.1	2.03	1.6	1.12	0.9	1.28	1	1.52	1.2
**8** NSC637712	3	1.92	1.84	0.96	10.86	5.7	17.67	9.2	3.02	1.6	2.92	1.5
Boceprevir*	n/a	0.148	0.217	1.8	0.743	4.7	7.227	65	0.100	0.7	1.546	5.8
Telaprevir*	n/a	0.150	0.886	10	1.470	10	20.326	105	0.046	0.4	ND	ND
Telaprevir¥	n/a	0.024	0.270	11.3	0.115	4.8	ND	ND	0.009	0.4	ND	ND

The IC_50_ values of the compounds were determined in the cleavage assays *in vitro* using the recombinant wild-type (WT) and mutant (V36M, R155K, A156T, D168A and V170A) NS3/4A constructs and Ac-DE-D(Edans)-EE-Abu-ψ-[COO]AS-K(Dabcyl)-NH_2_ as a substrate.

¥ The IC_50_ was directly measured *in vitro* using the purified recombinant WT and mutant NS3/4A constructs [75].

* The IC_50_ was measured using the mutant HCV replicon with luciferase readout [9], [14], [15]. FC, the fold change in the IC_50_ for the mutant compared to that the WT proteinase. ND, not determined, n/a, not applicable.

## Discussion

HCV is a causative agent of chronic liver disease worldwide with millions of infected patients at risk of developing significant morbidity and mortality. The HCV-encoded NS3/4A is essential for viral polyprotein processing and viral replication and has long been considered a promising drug target for pharmacological intervention in HCV-infected patients.

The NS3 proteinase represents the N-end, ∼180-residue, domain of the 631-residue NS3 protein. The C-end domain of NS3 encodes the ATP-dependent RNA helicase. In the course of polyprotein processing, NS3/4A cleaves the NS3-NS4A, NS4A-NS4B, NS4B-NS5A and NS5A-NS5B junctions and, as a result, generates the essential late viral non-structural proteins [49], [50]. The individual NS3 catalytic domain, however, is inactive. For its cleavage activity *in vitro* and *in vivo*, NS3 requires either the full-length NS4A cofactor or, at least, its 14-residue hydrophilic central portion [3], [51], [52]. NS4A is a 54 residue protein, with a hydrophobic N-terminus and a hydrophilic C-terminus. Following binding with NS4A, the NS3 domain is rearranged leading to the proper alignment of His-57, Asp-81, and Ser-139 of the catalytic triad [53], [54]. Because of its functional importance, NS3/4A is the prime anti-viral drug target [55], [56].

There is a consensus among scientists that therapeutic options and multi-component regiments should be expanded for HCV treatment [57], [58], [59]. In our search for the potential novel exosites in NS3/4A the targeting of which may lead to novel inhibitory scaffolds, we employed VLS using the 275,000 compound library of the Developmental Therapeutics Program (NCI/NIH) as a ligand source and the X-ray crystal structure of NS3/4A as a target. VLS was followed by extensive experimental *in vitro* and cell-based tests, and with the *in silico* SAR optimization of scaffolds. To perform both VLS and the *in silico* SAR optimization, we employed an unconventional, albeit highly efficient, protein-ligand docking technology developed by Q-MOL. This technology exploits protein flexibility for the identification of small molecule ligands, which are capable of interacting most efficiently with the most probable protein conformations in the folding energy landscape (a folding funnel) of a target protein [60], [61]. In the course of the Q-MOL protein-ligand docking simulations, the most probable protein conformations are implicitly evaluated for the individual ligands. Each dot in the VLS ranking curve relates not to the individual respective ligand alone but also to a theoretical protein conformation this ligand is likely to bind (Fig. 1). Because of its cumulative Gaussian nature, the Q-MOL VLS ranking curve reflects the Gaussian distribution of the protein conformations within their respective folding funnels. Because the structural diversity of protein conformations determines the ligand diversity and because certain protein conformations with a similar energy level may be distinct structurally, the ligands with the similar predicted binding energy may be structurally and chemically dissimilar. The Q-MOL VLS normally generates a range of the structurally different scaffolds for any flexible protein site, a result we achieved in our current study. Naturally, only a few of these scaffolds would exhibit the required amenable drug-like properties, including the required aqueous solubility, cytotoxicity, a low off-target activity and related parameters.

To increase a probability of scaffold hopping in our follow-on *in silico* SAR optimization efforts, we then used a chemical similarity parameter to generate a focused library around compounds **1**, **3** and **5.** The compounds in this focused library were then prioritized by docking to site 3 and the binding energy but not by chemical similarity. As a result of these efforts, we identified compounds **6, 7** and **8**. Because the compound core sub-structures are not always preserved in the remote analogs, compounds **6, 7** and **8** and the additional, moderately potent scaffolds we also identified are only remotely similar to the originating compounds **1, 3** and **5** (Tables 1 and S1). Taken together, our iterative *in silico* studies and enzymatic tests led us to the identification of several novel, nanomolar range inhibitory scaffolds which target the NS3/4A exosites. These novel scaffolds did not exhibit a significant level of cytotoxicity and off-target effects but they were capable of efficiently suppressing the NS3/4A functional activity *in vitro* and in cell-based assays.

Our cross-reactivity studies also dismissed the potential promiscuity of the compounds, which could be associated with their aggregation. The identification of these scaffolds confirms the efficiency of our VLS approach and also the presence of the exosites in the NS3/4A molecule that are, at least partially, outside the active site cavity of the proteinase and which could be probed using small molecule ligands. The most promising exosite we probed (docking site 3) appears to be similar to the one we recently identified in the structurally similar two-component NS2B-NS3 proteinase from West Nile virus [30], [62], [63]. According to our modeling studies (Fig. 5), compounds **4** and **7** to docking site 3 do not directly interact with the NS3/4A active site. In contrast, boceprevir directly interacts with the active site. The binding mode of boceprevir is highly similar to that of its derivative, (1R,2S,5S)-N-[(1S)-3-amino-1-(cyclobutylmethyl)-2,3-dioxopropyl]-6,6-dimethyl-3-(3-methyl-N-[(1-methylcyclohexyl) carbamoyl]-L-valyl)-3-azabicyclo[3.1.0]hexane-2-carboxamide (PDB 3LOX) [36]. The superimposition of compounds **4** and **7** with this boceprevir derivative in the PDB 3LOX structure suggests that there is a significant difference in the binding mode of boceprevir compared with the compounds we identified. This observation is in agreement with our *in vitro* inhibitory studies in the resistant NS3/4A mutants.

In turn, our modeling and biochemical data also suggest that certain novel compounds we tested, including compound **5**, overlap with the P2 site of NS3/4A and, as a result, with the P2 group of the α-ketoamide inhibitors (Fig. 5). In agreement and similar with cilupevir and ITMN-191 [15], [64] – the inhibitors with a sizable P2 substituent, the D168A mutation significantly affected the efficacy of compound **5** the pyrozolopyrimidine core of which interacts directly with Asp-168 (Table 2, Fig. 5). The potency of compounds **6**, **7** and **8**, however, was not significantly affected by the resistance mutations. Jointly with our modeling studies, these data imply that the binding of compounds **6**, **7**, and **8** does not likely involve the interactions with the P2 site of NS3/4A.

One of the promising inhibitory leads (compound **5**) could be transformed into an irreversible, covalent inhibitor to target non-catalytic, albeit essential, Cys-159. We believe that a possible mechanism of action of this next generation covalent inhibitor would be similar to that of AVL-192, a potent and specific covalent inhibitor that targets Cys-159 [65]. Cys-159, a non-catalytic amino acid that is present in all variants of NS3/4A, is targeted by AVL-192 that rapidly and completely silences NS3/4A.

Overall, our proof-of-principle work provides both conceptual support and methodology to probe the exosites of HCV NS3/4A with small molecule ligands for the follow-up rational structure-based inhibitor development and medicinal chemistry optimization of drug leads. We also believe that the *in silico* drug discovery approach employed in our study could be applied for the identification of inhibitors of other proteinases.

## Materials and Methods

### Reagents

Reagents were purchased from Sigma unless indicated otherwise. The FRET substrate Ac-DE-D(Edans)-EE-Abu-ψ-[COO]-AS-K(Dabsyl)-NH_2_, purified recombinant wild-type NS3/4A from HCV genotype 1b, strain HC-J4, and the mutants with the V36M, R155K, A156T, D168A and V170A resistance mutations were from AnaSpec (Fremont, CA). The NS3/4A construct represented the individual NS3 proteinase domain N-terminally fused with an NS4A co-factor and tagged with a 6xHis tag. NS3/4A was in active form and the pre-activation by pep-4A or pep4AK was not necessary [66]. Pyroglutamic acid-Arg-Thr-Lys-Arg-7-amino-4-methylcoumarin was from Peptides International (Louisville, KY). West Nile virus NS2B-NS3 proteinase (strain NY99) was expressed, purified and refolded to restore its functional activity as described earlier [67]. The pET15b-Den2-CF40-Gly-NS3pro 185 plasmid encoding the Dengue virus type 2 NS2B-NS3 protease was a kind gift of Dr. Subhash Vasudevan (Program in Emerging Infectious Diseases, DUKE-NUS Graduate Medical School, Singapore) [68]. DV NS2B-NS3 proteinase was expressed purified and refolded to restore its functional activity as described previously [69], [70]. The expression of the soluble C-terminally truncated human furin construct in Sf9 insect cells (an ovarian cell line from fall armyworm *Spodoptera frugiperda;* Invitrogen, Carlsbad, CA) infected with the recombinant baculovirus and purification of soluble furin from the medium were described earlier [71]. Original human hepatocarcinoma Huh7 cells were obtained from ATCC (Manassas, VA).

### Ligand and compound databases

The ligands and the compound databases in the SDF format were from “The NCI/DTP Open Chemical Repository” (http://dtp.nci.nih.gov). The compounds were >95% pure as certified by the supplier (NCI DTP Discovery Services). The NCI/DTP accession numbers (NSC) of the ligands are shown in Table S1. The ligands were dissolved in 100% dimethyl sulfoxide and stored at −20°C until use.

### Ligand docking sites

The protein-ligand docking simulations were performed using the NS3/4A crystal structure coordinates from PDB 3EYD [3]. The 2.5 Å resolution X-ray PDB 3EYD structure includes the A, B, C and D chains. Chains A and C represent the NS3 proteinase domain while chains B and D represent NS4A. Based on the PDB 3EYD structure, we specifically selected three individual ligand docking sites. Docking site 1 was defined as a 10 Å sphere centered at Val-26 of chain D. Chains B, C and D were then deleted from the structure and chain A alone was used in VLS. Docking site 2 was defined as a 10 Å sphere centered at Val-23 of chain B. Chains A, B and D were then deleted while chain C alone was used for VLS. Docking site 3 was defined as a 10 Å sphere centered at Val-167 of chain C. Chain C alone was used in VLS while chains A, B and D were deleted.

### Virtual ligand screening

VLS of a ∼275,000 compound library of the Developmental Therapeutics Program (DTP) NCI/NIH (http://dtp.nci.nih.gov) against the docking sites 1, 2 and 3 was performed using Q-MOL molecular modeling package (Q-MOL L.L.C., San Diego, CA, USA; www.q-mol.com) [30]. The Optimized Potential for Liquid Simulations (OPLS) all atom force field [72] is uniformly utilized within the Q-MOL program. The PDB 3EYD molecule preparation included adding of hydrogen atoms and the assignment of the standard OPLS atom types. To increase the speed of calculations and to incorporate implicitly the flexibility of NS3/4A, the PDB 3EYD structure was treated as a set of grid-based potentials accounting for the relevant protein – ligand interactions. The ligands were docked into the grid-based potentials using a Monte Carlo simulation in the internal coordinate space as implemented in the Q-MOL program. The preparation of each ligand for docking simulation initially included an automatic OPLS atom type assignment and conversion of the two-dimensional sketch-like models (input as the MDL MOL format) into the three-dimensional molecular models. The full-atom ligand structure was then minimized using the Q-MOL small molecule minimization protocol. The protocol combines minimization in both internal and Cartesian coordinates to properly optimize the rotable bonds of a small molecule. The ligands, which failed minimization, were not included into the follow-up VLS. To increase the performance of VLS, the NCI compound database was converted into a binary space partitioning (BSP) tree-like structure [30]. Prior to VLS, polyphenols and compounds with either a molecular mass below 220 Da or chlorine atoms attached to aliphatic carbons were filtered out from the NCI database. Because of the stochastic nature of the Q-MOL docking protocol, each ligand was docked at least three times. To differentiate between true and false-positive binders, Q-MOL employs a proprietary protein-ligand binding energy evaluation function. This function is based on the re-parameterized OPLS force field, and, in addition to the protein-ligand interactions, it accounts for the internal energy change of the docked ligands. The identified hits were ranked according to their binding energy and then visually inspected to discard those with high molecular weight, high symmetry or heavily halogenated compounds. The resulting hits with the lowest binding energy were selected for our further studies.

### *In silico* Structure-Activity-Relationship (SAR) optimization

The chemical structures of the initial *in vitro* validated hits **1**, **3** and **5** (Table S1) were used as seeds for searching of the NCI database and then for building a focused, 750 compound sub-library (250 derivatives per each initial hit). The Tanimoto distance, calculated using the proprietary Q-MOL molecular fingerprints, was used as a chemical similarity measure to identify the structures similar to the initial hits. The original validated hits were also included into the sub-library as references. The sub-library was re-docked into the docking site 3 of NS3/4A using our VLS protocol. From the 750 selected structures 85 failed minimization and were discarded. The 100 best predicted binders with the lowest binding energy were visually inspected and 20 available compounds were ordered from the NCI/DTP for *in vitro* activity testing.

### Structure modeling

The predicted binding modes of the ligands were built using the full-atom flexible protein-ligand docking in the internal coordinates. The initial ligand conformations were taken from the VLS experiments. The protein-ligand complexes were then globally optimized in the OPLS force field using the Monte Carlo simulation. The protein ψ, φ and χ angles of all of the amino acid residues, which are within a 7.5 Å distance from ligand atoms, were allowed to change while the positional and rotable torsion variables of a ligand molecule were unfixed.

### Proteinase activity assays with fluorescent peptide

The NS3/4A activity assay was performed at 37°C in triplicate in wells of a 96-well plate in 0.2 ml 50 mM HEPES, pH 7.5, containing 100 mM NaCl, 10 mM DTT, 20% glycerol, the Ac-DE-D(Edans)-EE-Abu-ψ-[COO]-AS-K(Dabsyl)-NH_2_ substrate (10 µM) and the NS3/4A enzyme (10 nM). Reaction velocity was monitored continuously at λ_ex_ = 355 nm and λ_em_ = 500 nm for 10 min using a Spectramax Gemini EM fluorescence spectrophotometer (Molecular Devices, Sunnyvale, CA) and the initial rate of reaction v was recorded for each reaction. There was no significant additional absorption by compounds **1–8** we identified, which might interfere with NS3/4A assay. Compound stock solutions (10 mM) were made in 100% DMSO. Stocks were then diluted using the assay buffer. As a result, the final DMSO concentration was always below 1% in the assay reactions.

The activity of furin and both WNV and DV2 NS2B-NS3 proteinases (10 nM each) was measured in triplicate in 0.2 ml 20 mM Tris-HCl buffer, pH 8.0, containing 20% glycerol and 0.005% Brij 35 (for furin – 100 mM HEPES, pH 7.5, containing 1 mM CaCl_2_, 1 mM β-mercaptoethanol and 0.005% Brij 35) and pyroglutamic acid-Arg-Thr-Lys-Arg-7-amino-4-methylcoumarin (10 µM) as a substrate (λ_ex_ = 360 nm; λ_em_ = 465 nm) as described earlier [30].

To measure the inhibitory potency of the compounds, their increasing concentrations were pre-incubated with the individual proteinases in the corresponding buffer for 30 min at ambient temperature. At least, 7–9 different concentrations were used for each inhibitor. The residual activity of the enzymes was then measured as above. The IC_50_ values of the inhibitory compounds were calculated by determining the concentrations of the compounds needed to inhibit 50% of the enzyme activity against the peptide substrate. GraphPad Prism was used as fitting software.

### HCV replicon and cell toxicity assays

Both cell toxicity and HCV replicon inhibition assays were performed in triplicate in wells of a 96-well flat-bottom, white-wall plates (E&K Scientific, Santa Clara, CA) by the Drug Development Division, Southern Research Institute (SRI; Birmingham, AL). The human hepatocarcinoma cell line Huh7 ET (luc-ubi-neo/ET) that exhibits a sub-genomic HCV RNA replicon with a stable firefly luciferase reporter was used in both assay types. Prior to the assays, sub-confluent Huh7 ET cultures were seeded in wells of a 96-well plate. Following a 24 h incubation at 37°C in a 5% CO_2_ incubator, increasing concentrations of the compounds were added to the wells and incubation was continued for an additional 72 h. The final concentration of DMSO in the cell assays did not exceeded 1% for the highest concentration of the compounds. Human interferon α2b was used as a control. The cellular replicon-derived luciferase activity was measured using the Steady-Glo Luciferase Assay System (Promega, Fitchburg, WI) and a fluorescence spectrophotometer. Cytotoxicity of compounds was measured using the CytoTox-ONE Homogeneous Membrane Integrity Assay (Promega). The IC_50_ and IC_90_ values of the compounds (compound concentrations inhibiting the replicon by 50% and 90%, respectively) as well as CC_50_ and CC_90_ (compound concentration decreasing cell viability by 50% and 90%, respectively) were calculated using GraphPad Prism.

## Supporting Information

Table S1 **Selected inhibitors of HCV NS3/4A.** Only the IC_50_ values below 10 µM are shown. +, the IC_50_ value of the inhibitor is either in the 10–100 µM or 100–1000 µM range. The HCV NS3/4A activity was measured using the Ac-DE-D(Edans)-EE-Abu-ψ-[COO]AS-K(Dabcyl)-NH_2_ substrate. SAR indicates the compounds to docking site 3 which have been identified through our SAR efforts.
(DOC)
