# Supplementary material for: Probing of Exosites Leads to Novel Inhibitor Scaffolds of HCV NS3/4A Proteinase
Source: PLoS One. 2012 Jul 2;7(7):e40029. doi: 10.1371/journal.pone.0040029 (PMC3388044; doi:10.1371/journal.pone.0040029)
Supplement: Table S1 — Selected inhibitors of HCV NS3/4A. Only the IC50 values below 10 µM are shown. +, the IC50 value of the inhibitor is either in the 10–100 µM or 100–1000 µM range. The HCV NS3/4A activity was measured using the Ac-DE-D(Edans)-EE-Abu-ψ-[COO]AS-K(Dabcyl)-NH2 substrate. SAR indicates the compounds to docking site 3 which have been identified through our SAR efforts. (DOC) [file pone.0040029.s001.doc]

| Compound | NCI  identifier | PubChem  CID identifier | Structure | NS3/4A  docking site | IC_50_, µM | | | |
| --- | --- | --- | --- | --- | --- | --- | --- | --- |
|  |  |  |  |  | < 1 | 1 - 10 | 10 -100 | 100-1000 |
| **1** | NSC704342 | 397060 | [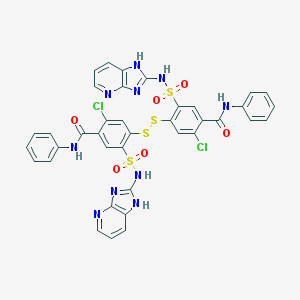](javascript:%20void%20window.open('../image/structurefly.cgi?cid=397060&width=400&height=400',%20'StructureFly',%20'resizable=yes,%20scrollbars=yes,%20WIDTH=620,%20HEIGHT%20=%20620')) | 3 | 0.183 |  |  |  |
| **2** | NSC713288 | 400782 | [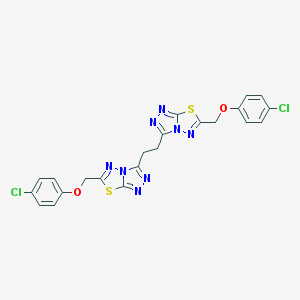](javascript:%20void%20window.open('../image/structurefly.cgi?cid=400782&width=400&height=400',%20'StructureFly',%20'resizable=yes,%20scrollbars=yes,%20WIDTH=620,%20HEIGHT%20=%20620')) | 2 | 0.3 |  |  |  |
| **3** | NSC724526 | 5473230 | [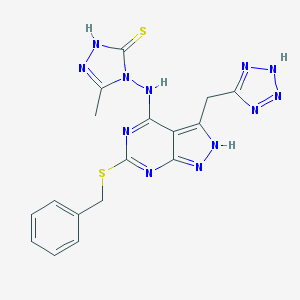](javascript:%20void%20window.open('../image/structurefly.cgi?cid=5473230&width=400&height=400',%20'StructureFly',%20'resizable=yes,%20scrollbars=yes,%20WIDTH=620,%20HEIGHT%20=%20620')) | 3 | 0.36 |  |  |  |
| **4** | NSC320254 | 5479817 | 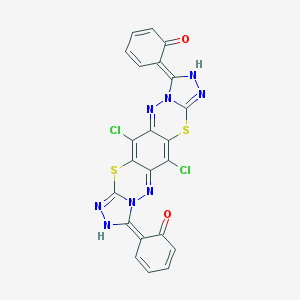 | 3 – SAR | 0.36 |  |  |  |
| **5** | NSC724527 | 406453 | [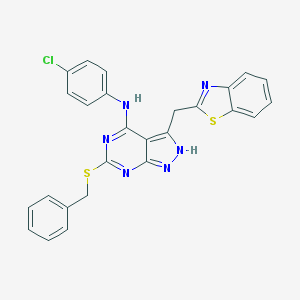](javascript:%20void%20window.open('../image/structurefly.cgi?cid=406453&width=400&height=400',%20'StructureFly',%20'resizable=yes,%20scrollbars=yes,%20WIDTH=620,%20HEIGHT%20=%20620')) | 3 | 0.38 |  |  |  |
| **6** | NSC724525 | 5473229 | [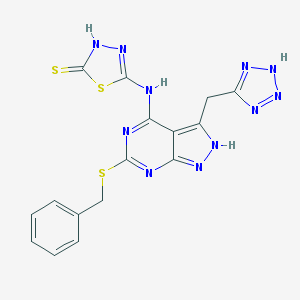](javascript:%20void%20window.open('../image/structurefly.cgi?cid=5473229&width=400&height=400',%20'StructureFly',%20'resizable=yes,%20scrollbars=yes,%20WIDTH=620,%20HEIGHT%20=%20620')) | 3 – SAR | 0.95 |  |  |  |
| **7** | NSC716899 | 5472203 | [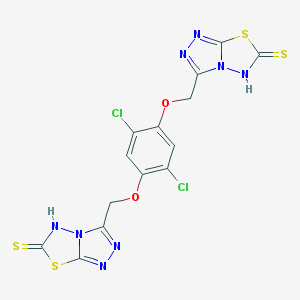](javascript:%20void%20window.open('../image/structurefly.cgi?cid=5472203&width=400&height=400',%20'StructureFly',%20'resizable=yes,%20scrollbars=yes,%20WIDTH=620,%20HEIGHT%20=%20620')) | 3 – SAR |  | 1.25 |  |  |
| **8** | NSC637712 | 367773 | [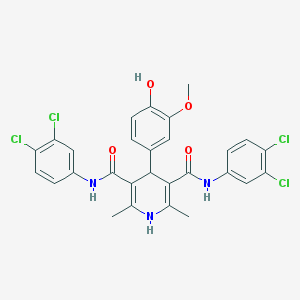](javascript:%20void%20window.open('../image/structurefly.cgi?cid=367773&width=400&height=400',%20'StructureFly',%20'resizable=yes,%20scrollbars=yes,%20WIDTH=620,%20HEIGHT%20=%20620')) | 3 – SAR |  | 1.92 |  |  |
| **9** | NSC217338 | 311779 | [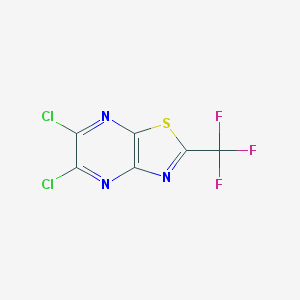](javascript:%20void%20window.open('../image/structurefly.cgi?cid=311779&width=400&height=400',%20'StructureFly',%20'resizable=yes,%20scrollbars=yes,%20WIDTH=620,%20HEIGHT%20=%20620')) | 3 |  | 4.5 |  |  |
| **10** | NSC725733 | 11039287 | [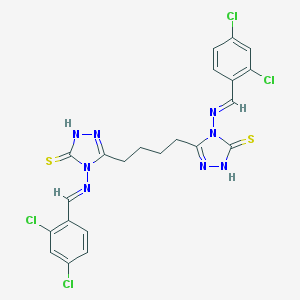](javascript:%20void%20window.open('../image/structurefly.cgi?cid=11039287&width=400&height=400',%20'StructureFly',%20'resizable=yes,%20scrollbars=yes,%20WIDTH=620,%20HEIGHT%20=%20620')) | 3 – SAR |  | 5.25 |  |  |
| **11** | NSC728074 | 23635643 | [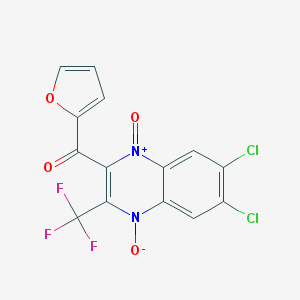](javascript:%20void%20window.open('../image/structurefly.cgi?cid=23635643&width=400&height=400',%20'StructureFly',%20'resizable=yes,%20scrollbars=yes,%20WIDTH=620,%20HEIGHT%20=%20620')) | 3 |  | 6.5 |  |  |
| **12** | NSC1456 | 219783 | [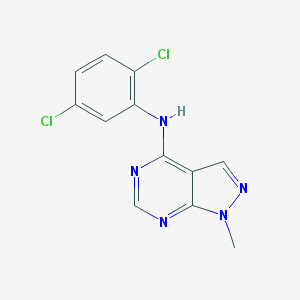](javascript:%20void%20window.open('../image/structurefly.cgi?cid=219783&width=400&height=400',%20'StructureFly',%20'resizable=yes,%20scrollbars=yes,%20WIDTH=620,%20HEIGHT%20=%20620')) | 3 |  |  | + |  |
| **13** | NSC48873 | 241475 | [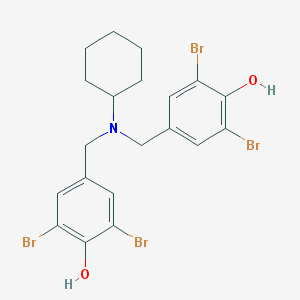](javascript:%20void%20window.open('../image/structurefly.cgi?cid=241475&width=400&height=400',%20'StructureFly',%20'resizable=yes,%20scrollbars=yes,%20WIDTH=620,%20HEIGHT%20=%20620')) | 1 |  |  | + |  |
| **14** | NSC51648 | 54609858 | 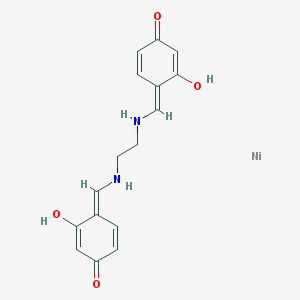 | 1 |  |  | + |  |
| **15** | NSC56529 | 245037 | 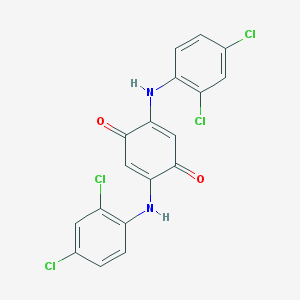 | 2 |  |  | + |  |
| **16** | NSC68243 | 249671 | [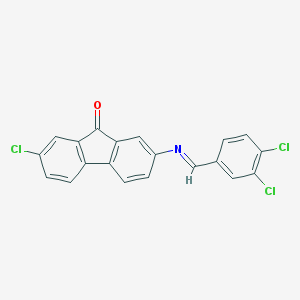](javascript:%20void%20window.open('../image/structurefly.cgi?cid=249671&width=400&height=400',%20'StructureFly',%20'resizable=yes,%20scrollbars=yes,%20WIDTH=620,%20HEIGHT%20=%20620')) | 2 |  |  | + |  |
| **17** | NSC106121 | 6868395 | 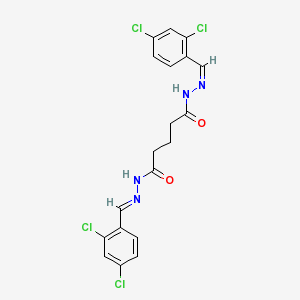 | 2 |  |  | + |  |
| **18** | NSC177407 | 5383615 | [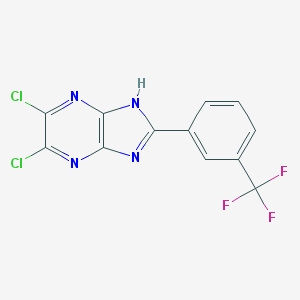](javascript:%20void%20window.open('../image/structurefly.cgi?cid=5383615&width=400&height=400',%20'StructureFly',%20'resizable=yes,%20scrollbars=yes,%20WIDTH=620,%20HEIGHT%20=%20620')) | 3 |  |  | + |  |
| **19** | NSC191876 | 303487 | 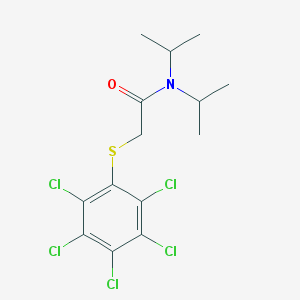 | 2 |  |  | + |  |
| **20** | NSC201628 | 5716180 | 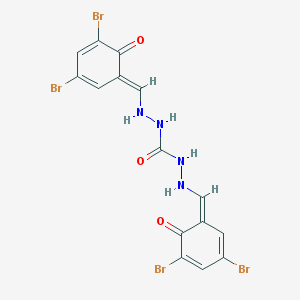 | 1 |  |  | + |  |
| **21** | NSC204225 | 306542 | 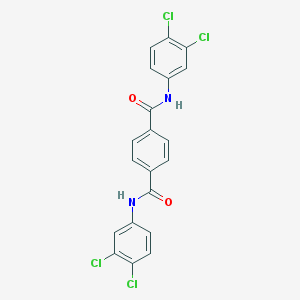 | 2 |  |  | + |  |
| **22** | NSC204553 | 5200560 | 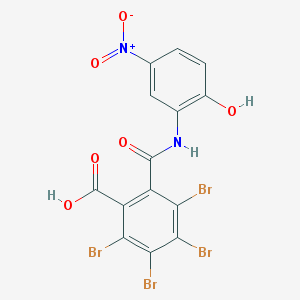 | 1 |  |  | + |  |
| **23** | NSC217041 | 311701 | 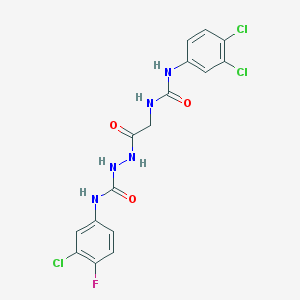 | 2 |  |  | + |  |
| **24** | NSC323897 | 358743 | 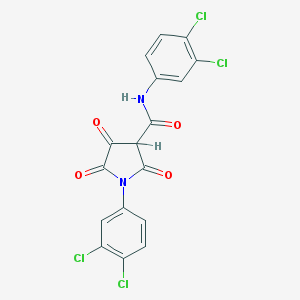 | 2 |  |  | + |  |
| **25** | NSC343061 | 434132 | 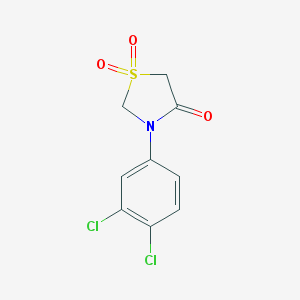 | 2 |  |  | + |  |
| **26** | NSC346849 | 335760 | 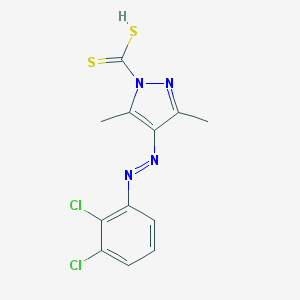 | 3 |  |  | + |  |
| **27** | NSC346851 | 335762 | 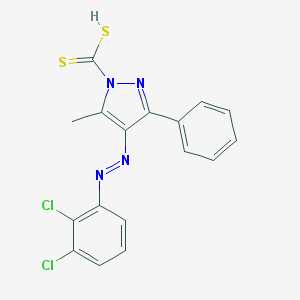 | 3 |  |  | + |  |
| **28** | NSC347771 | 5116139 | 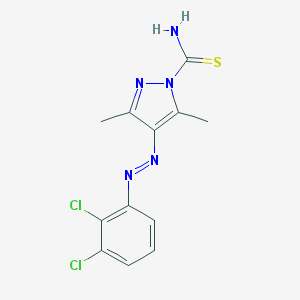 | 3 |  |  | + |  |
| **29** | NSC348917 | 336000 | 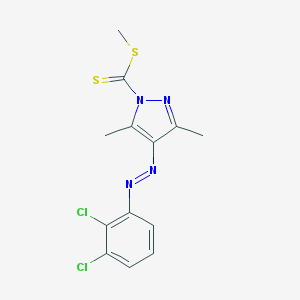 | 3 |  |  | + |  |
| **30** | NSC371810 | 340735 | [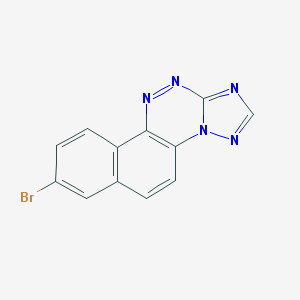](javascript:%20void%20window.open('../image/structurefly.cgi?cid=340735&width=400&height=400',%20'StructureFly',%20'resizable=yes,%20scrollbars=yes,%20WIDTH=620,%20HEIGHT%20=%20620')) | 3 – SAR |  |  | + |  |
| **31** | NSC633361 | 365566 | [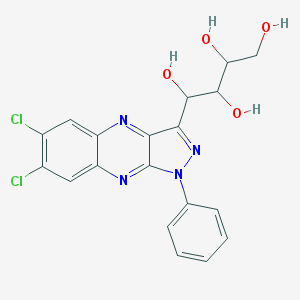](javascript:%20void%20window.open('../image/structurefly.cgi?cid=365566&width=400&height=400',%20'StructureFly',%20'resizable=yes,%20scrollbars=yes,%20WIDTH=620,%20HEIGHT%20=%20620')) | 2 |  |  | + |  |
| **32** | NSC656343 | 5812862 | [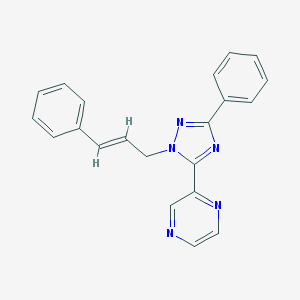](javascript:%20void%20window.open('../image/structurefly.cgi?cid=5812862&width=400&height=400',%20'StructureFly',%20'resizable=yes,%20scrollbars=yes,%20WIDTH=620,%20HEIGHT%20=%20620')) | 1 |  |  | + |  |
| **33** | NSC704335 | 397053 | [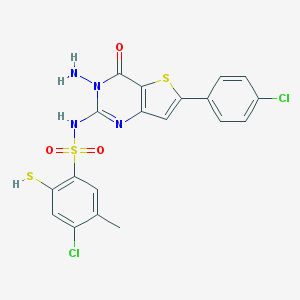](javascript:%20void%20window.open('../image/structurefly.cgi?cid=397053&width=400&height=400',%20'StructureFly',%20'resizable=yes,%20scrollbars=yes,%20WIDTH=620,%20HEIGHT%20=%20620')) | 3 |  |  | + |  |
| **34** | NSC725244 | 406914 | [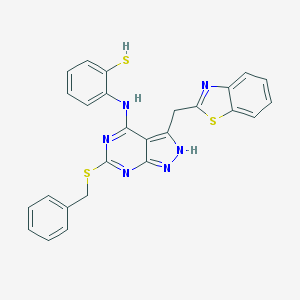](javascript:%20void%20window.open('../image/structurefly.cgi?cid=406914&width=400&height=400',%20'StructureFly',%20'resizable=yes,%20scrollbars=yes,%20WIDTH=620,%20HEIGHT%20=%20620')) | 3 |  |  | + |  |
| **35** | NSC725740 | 11734970 | [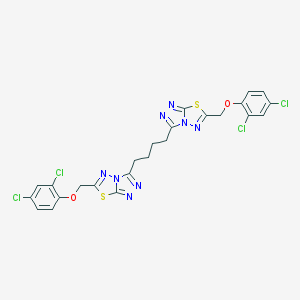](javascript:%20void%20window.open('../image/structurefly.cgi?cid=11734970&width=400&height=400',%20'StructureFly',%20'resizable=yes,%20scrollbars=yes,%20WIDTH=620,%20HEIGHT%20=%20620')) | 2 |  |  | + |  |
| **36** | NSC128940 | 278972 | [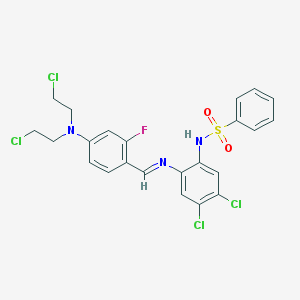](javascript:%20void%20window.open('../image/structurefly.cgi?cid=278972&width=400&height=400',%20'StructureFly',%20'resizable=yes,%20scrollbars=yes,%20WIDTH=620,%20HEIGHT%20=%20620')) | 3 – SAR |  |  | + |  |
| **37** | NSC128947 | 6245503 | [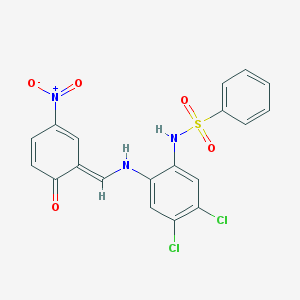](javascript:%20void%20window.open('../image/structurefly.cgi?cid=6245503&width=400&height=400',%20'StructureFly',%20'resizable=yes,%20scrollbars=yes,%20WIDTH=620,%20HEIGHT%20=%20620')) | 3 – SAR |  |  | + |  |
| **38** | NSC156842 | 291540 | 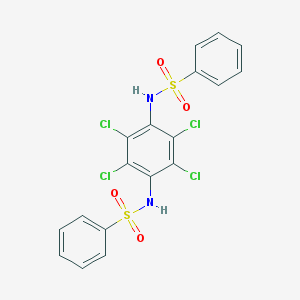 | 3 – SAR |  |  | + |  |
| **39** | NSC640984 | 369253 | [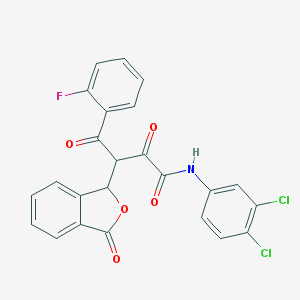](javascript:%20void%20window.open('../image/structurefly.cgi?cid=369253&width=400&height=400',%20'StructureFly',%20'resizable=yes,%20scrollbars=yes,%20WIDTH=620,%20HEIGHT%20=%20620')) | 3 – SAR |  |  | + |  |
| **40** | NSC724524 | 406450 | [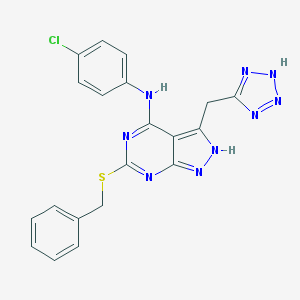](javascript:%20void%20window.open('../image/structurefly.cgi?cid=406450&width=400&height=400',%20'StructureFly',%20'resizable=yes,%20scrollbars=yes,%20WIDTH=620,%20HEIGHT%20=%20620')) | 3 |  |  | + |  |
| **41** | NSC725244 | 406914 | [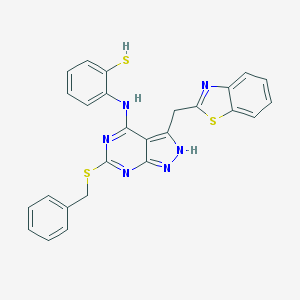](javascript:%20void%20window.open('../image/structurefly.cgi?cid=406914&width=400&height=400',%20'StructureFly',%20'resizable=yes,%20scrollbars=yes,%20WIDTH=620,%20HEIGHT%20=%20620')) | 3 – SAR |  |  | + |  |
| **42** | NSC19125 | 227518 | [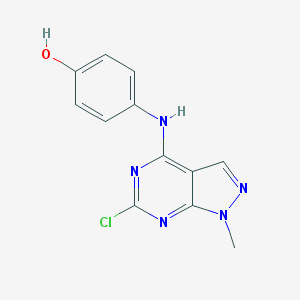](javascript:%20void%20window.open('../image/structurefly.cgi?cid=227518&width=400&height=400',%20'StructureFly',%20'resizable=yes,%20scrollbars=yes,%20WIDTH=620,%20HEIGHT%20=%20620')) | 1 |  |  |  | + |
| **43** | NSC34412 | 95768 | [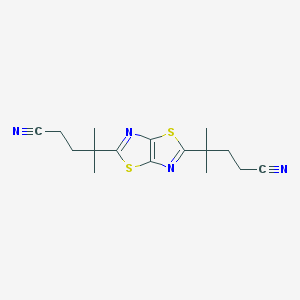](javascript:%20void%20window.open('../image/structurefly.cgi?cid=95768&width=400&height=400',%20'StructureFly',%20'resizable=yes,%20scrollbars=yes,%20WIDTH=620,%20HEIGHT%20=%20620')) | 3 |  |  |  | + |
| **44** | NSC129410 | 97522 | [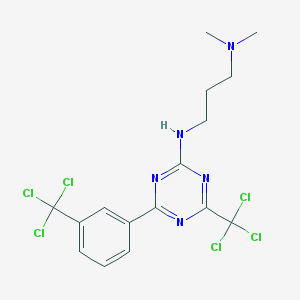](javascript:%20void%20window.open('../image/structurefly.cgi?cid=97522&width=400&height=400',%20'StructureFly',%20'resizable=yes,%20scrollbars=yes,%20WIDTH=620,%20HEIGHT%20=%20620')) | 2 |  |  |  | + |
| **45** | NSC165919 | 5383345 | [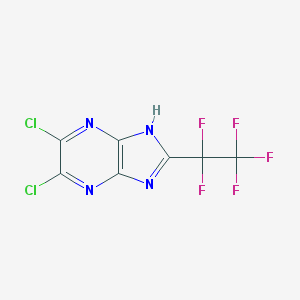](javascript:%20void%20window.open('../image/structurefly.cgi?cid=5383345&width=400&height=400',%20'StructureFly',%20'resizable=yes,%20scrollbars=yes,%20WIDTH=620,%20HEIGHT%20=%20620')) | 2 |  |  |  | + |
| **46** | NSC166547 | 296409 | [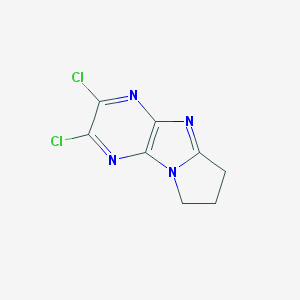](javascript:%20void%20window.open('../image/structurefly.cgi?cid=296409&width=400&height=400',%20'StructureFly',%20'resizable=yes,%20scrollbars=yes,%20WIDTH=620,%20HEIGHT%20=%20620')) | 2 |  |  |  | + |
| **47** | NSC182397 | 5383684 | [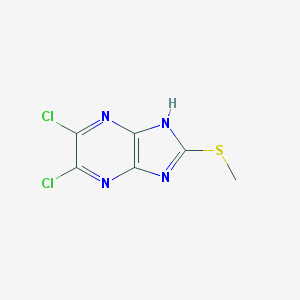](javascript:%20void%20window.open('../image/structurefly.cgi?cid=5383684&width=400&height=400',%20'StructureFly',%20'resizable=yes,%20scrollbars=yes,%20WIDTH=620,%20HEIGHT%20=%20620')) | 3 |  |  |  | + |
| **48** | NSC217916 | 5358028 | [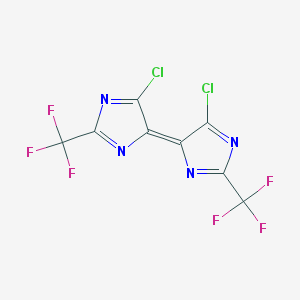](javascript:%20void%20window.open('../image/structurefly.cgi?cid=5358028&width=400&height=400',%20'StructureFly',%20'resizable=yes,%20scrollbars=yes,%20WIDTH=620,%20HEIGHT%20=%20620')) | 3 |  |  |  | + |
| **49** | NSC305774 | 24198821 | [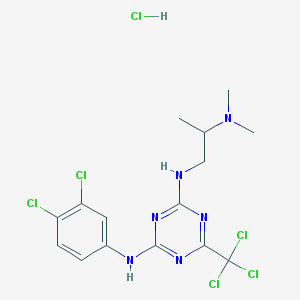](javascript:%20void%20window.open('../image/structurefly.cgi?cid=24198821&width=400&height=400',%20'StructureFly',%20'resizable=yes,%20scrollbars=yes,%20WIDTH=620,%20HEIGHT%20=%20620')) | 2 |  |  |  | + |
| **50** | NSC642653 | 5466778 | [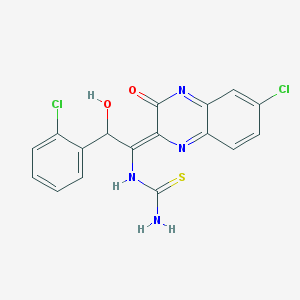](javascript:%20void%20window.open('../image/structurefly.cgi?cid=5466778&width=400&height=400',%20'StructureFly',%20'resizable=yes,%20scrollbars=yes,%20WIDTH=620,%20HEIGHT%20=%20620')) | 3 |  |  |  | + |
| **51** | NSC656337 | 375971 | [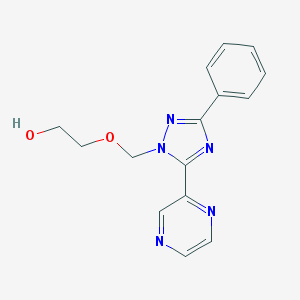](javascript:%20void%20window.open('../image/structurefly.cgi?cid=375971&width=400&height=400',%20'StructureFly',%20'resizable=yes,%20scrollbars=yes,%20WIDTH=620,%20HEIGHT%20=%20620')) | 1 |  |  |  | + |
| **52** | NSC156843 | 291541 | [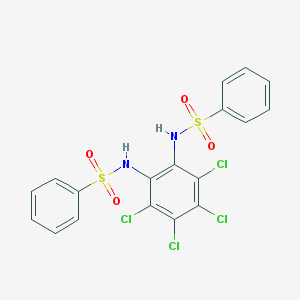](javascript:%20void%20window.open('../image/structurefly.cgi?cid=291541&width=400&height=400',%20'StructureFly',%20'resizable=yes,%20scrollbars=yes,%20WIDTH=620,%20HEIGHT%20=%20620')) | 3 - SAR |  |  |  | + |
| **53** | NSC298138 | 326507 | 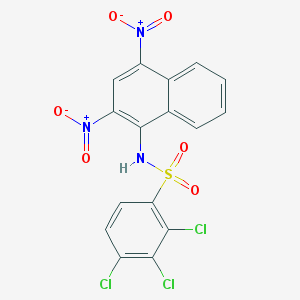 | 3 – SAR |  |  |  | + |
| **54** | NSC635536 | 5388386 | [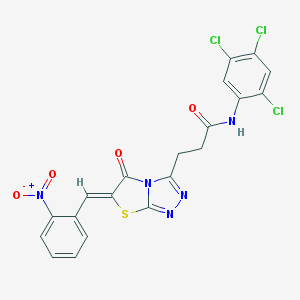](javascript:%20void%20window.open('../image/structurefly.cgi?cid=5388386&width=400&height=400',%20'StructureFly',%20'resizable=yes,%20scrollbars=yes,%20WIDTH=620,%20HEIGHT%20=%20620')) | 3 – SAR |  |  |  | + |
| **55** | NSC645881 | 371552 | [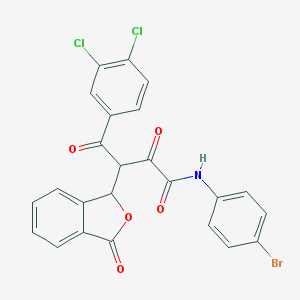](javascript:%20void%20window.open('../image/structurefly.cgi?cid=371552&width=400&height=400',%20'StructureFly',%20'resizable=yes,%20scrollbars=yes,%20WIDTH=620,%20HEIGHT%20=%20620')) | 3 – SAR |  |  |  | + |
| **56** | NSC658259 | 377043 | [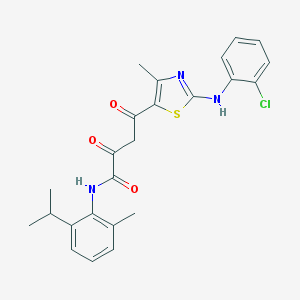](javascript:%20void%20window.open('../image/structurefly.cgi?cid=377043&width=400&height=400',%20'StructureFly',%20'resizable=yes,%20scrollbars=yes,%20WIDTH=620,%20HEIGHT%20=%20620')) | 3 – SAR |  |  |  | + |
| **57** | NSC716350 | 402419 | 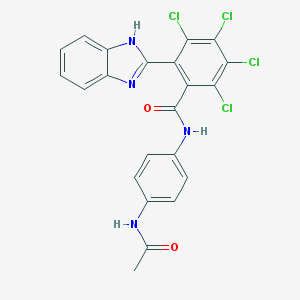 | 3 – SAR |  |  |  | + |
| **58** | NSC716352 | 402421 | [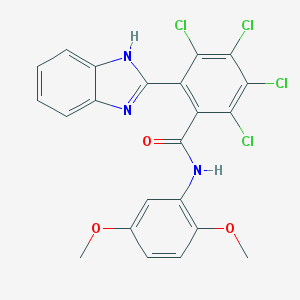](javascript:%20void%20window.open('../image/structurefly.cgi?cid=402421&width=400&height=400',%20'StructureFly',%20'resizable=yes,%20scrollbars=yes,%20WIDTH=620,%20HEIGHT%20=%20620')) | 3 – SAR |  |  |  | + |
| **59** | NSC716891 | 5472201 | [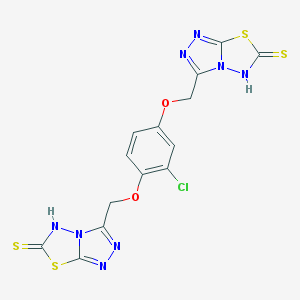](javascript:%20void%20window.open('../image/structurefly.cgi?cid=5472201&width=400&height=400',%20'StructureFly',%20'resizable=yes,%20scrollbars=yes,%20WIDTH=620,%20HEIGHT%20=%20620')) | 3 – SAR |  |  |  | + |
| **60** | NSC725845 | 24202746 | 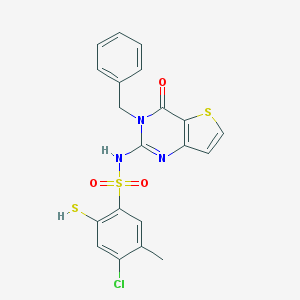 | 3 - SAR |  |  |  | + |
